# Antioxidant Activity and Total Phenolic and Flavonoid Contents of *Hieracium pilosella* L. Extracts

**DOI:** 10.3390/s90705702

**Published:** 2009-07-16

**Authors:** Ljiljana Stanojević, Mihajlo Stanković, Vesna Nikolić, Ljubiša Nikolić, Dušica Ristić, Jasna Čanadanovic-Brunet, Vesna Tumbas

**Affiliations:** 1 University of Nis, Faculty of Technology, Bulevar Oslobodjenja 124, 16000 Leskovac, Serbia; E-Mails: mstankovic_99@yahoo.com (M.S.); nvesna@yahoo.com (V.N.); nljubisa@yahoo.com (L.N.); dusica.aleksandar@gmail.com (D.R.); 2 University of Novi Sad, Faculty of Technology, Bulevar Cara Lazara 1, 21000 Novi Sad, Serbia; E-Mails: brunet_j@EUnet.yu (J.C-B.); vesnat@uns.ns.ac.yu (V.T)

**Keywords:** *Hieracium pilosella* L. (*Asteraceae*), antioxidant activity, extraction, total phenolic content, total flavonoids, HPLC determination

## Abstract

The antioxidant activity of water, ethanol and methanol *Hieracium pilosella* L. extracts is reported. The antioxidative activity was tested by spectrophotometrically measuring their ability to scavenge a stable DPPH^•^ free radical and a reactive hydroxyl radical trapped by DMPO during the Fenton reaction, using the ESR spectroscopy. Total phenolic content and total flavonoid content were evaluated according to the Folin-Ciocalteu procedure, and a colorimetric method, respectively. A HPLC method was used for identification of some phenolic compounds (chlorogenic acid, apigenin-7-*O*-glucoside and umbelliferone). The antioxidant activity of the investigated extracts slightly differs depending on the solvent used. The concentration of 0.30 mg/mL of water, ethanol and methanol extract is less effective in scavenging hydroxyl radicals (56.35, 58.73 and 54.35%, respectively) in comparison with the DPPH^•^ radical scavenging activity (around 95% for all extracts). The high contents of total phenolic compounds (239.59–244.16 mg GAE/g of dry extract) and total flavonoids (79.13–82.18 mg RE/g of dry extract) indicated that these compounds contribute to the antioxidative activity.

## 1. Introduction

Free radicals contribute to more than one hundred disorders in humans including atherosclerosis, arthritis, ischemia and repercussion injury of many tissues, a central nervous system injury, gastritis and cancer [1–4]. Due to environmental pollutants, radiation, chemicals, toxins, deep fries and spicy foods as well as physical stress, free radicals cause depletion of the immune system antioxidants, the change in gene expression and induce abnormal proteins. The oxidation process is one of the most important routs for producing free radicals in food, drugs, and even living systems [1,5,6].

Antioxidants are important species which possess the ability of protecting organisms from damage caused by free radical-induced oxidative stress [7]. The antioxidant activity of phenolics is mainly due to their redox properties, which allow them to act as reducing agents, hydrogen donors, singlet oxygen quenchers and metal chelators [7–9]. A number of synthetic antioxidants such as butylated hydroxyanisole (BHA) and butylated hydroxytoluene (BHT) have been extensively added to foodstuffs, although their use has begun to be questioned because of their toxicity [10,11], so there is considerable interest in preventive medicine and in the food industry in the development of natural antioxidants obtained from botanical sources, especially herbal plants [12].

Medicinal plants have been used for centuries as remedies for human diseases because they contain components of therapeutic value [9,13–14]. Moreover, the increasing use of plant extracts in the food, cosmetic and pharmaceutical industries suggests that, in order to find active compounds, a systematic study of medicinal plants is very important [7,13].

The large genus *Hieracium* L. consists of over 1,000 species. Some species, such as *H. pilosella*, *H. auranticum* and *H. murorum*, are used in traditional European medicines as they display diuretic and anti-inflammatory effects [15].

*Hieracium pilosella* L. (Family: *Asteraceae*) is a perennial herbaceous plant. It is widely spread in mountain and foothill pastures, in oak woods and underbrush areas. It is mainly used as a traditional medicine for bronchitis, bronchial asthma, edema, and as an ointment for wound healing. It is especially recommended for intensifying urination and eliminating slime, sand and small stones from the urinary tract and the kidneys [16,17]. Because of its medicinal value, it has been used in traditional Serbian medicine for centuries [17].

The phenolic components most frequently represented in methanol extracts from all *Hieracium* species are: chlorogenic acid, caffeic acid, and umbelliferone, and among these, umbelliferone is the most active one [18–20]. The phenolic acids and flavonoids present in the plants are natural antioxidants [7,21–23]. They also have anti-mutagenic and anti-cancerogenic properties [24], cardio-protective [25], anti-inflammatory [11] and antimicrobial activity [16,26,27].

Chlorogenic acid is a highly valuable natural polyphenolic compound used in medicine and industry. Chlorogenic acid is used as an additive in various beverages, cosmetics, tea products, and foods as well as in medical substances. Chlorogenic acid has antibacterial and antiviral properties, and it is a natural antioxidant and anticancer agent. The current commercial sources of chlorgenic acids are from plant extracts of plants such as *Lonicera japonica Thunb* and *Eucommia ulmoides Oliver*. These sources are generally limited and therefore expensive [28].

In this paper, the antioxidant activity of aqueous, ethanolic and methanolic extracts from *Hieracium pilosella* L. from Southeast Serbia was investigated. DPPH radical scavenging activity of different extracts was investigated spectrophotometrically. A free radical scavenging activity on the reactive hydroxyl radical formed in the Fenton reaction was investigated by ESR spectroscopy. The total phenolic and flavonoid contents in the extracts was determined and correlated with the antioxidant activity. The total content of the investigated phenolic components (chlorogenic acid, umbelliferone and apigenin-7-*O*-glucoside) was determined by HPLC analysis.

## 2. Results and Discussion

The yields of the extracts obtained per 100 g of dry plant material with the different solvents are given in Table 1.

The highest yield of the extract (44.0 g/100 g of dry plant material was obtained by extraction with 50% v/v ethanol. The yield of ethanolic extract was higher than yield of aqueous and methanolic extract for 13.20 and 3.80% respectively.

Based on HPLC analysis and the calibration curves of the standard samples, the contents of the investigated compounds were determined in all the extracts (Table 2).

The highest quantities of chlorogenic acid and apigenin-7-*O*-glucoside (21.60 and 0.25 g/100 g of dry plant material, respectively) were extracted using 50% ethanol, and the highest yield of umbelliferone was obtained using water (0.65 g/100 g dry plant material). The quantity of chlorogenic acid in dry extract extracted using 50% ethanol is higher than the quantity of chlorogenic acid extracted using water and 80% methanol, respectively for 7.54 and 11.10%, respectively. The quantity of umbeliferone in dry extract extracted using water is higher than the quantities extracted using 50% ethanol and 80% methanol (for 57.4 and 19.53%, respectively). The quantity of apigenin-7-*O*-glucoside in dry extract extracted using 50% ethanol is higher than quantity of umbeliferone extracted using water and 80% methanol, respectively (63.8 and 72.40%). Chlorogenic acid was detected in the highest quantities in all the extracts. Considering a high content of this component in the extracts *H. pilosella* L., this plant can represent a potential natural resource.

The extracts obtained by different solvents were subjected to screening for their possible antioxidant activity. Four complementary test systems, namely hydroxyl radical scavenging, DPPH free radical-scavenging, total phenolic compounds, and total flavonoids content, were used for this purpose.

The antioxidant activity of the aqueous, ethanolic and methanolic extracts of *H. pilosella* L. was investigated by the ability of the extract to scavenge hydroxyl radicals. This is very important because of the fact that hydroxyl radicals were mentioned as the major active oxygen species causing lipid oxidation [7]. Using the spin trap, such as DMPO, it is possible to convert reactive hydroxyl radicals to stable nitroxide radicals (DMPO-OH adducts) with spectral hyperfine splittings that reflect the nature and structure of these radicals. The relative intensity of free radical formation can be determined because the ESR spectroscopy signal is directly related to the concentration of spin adducts.

**Figure 1.**
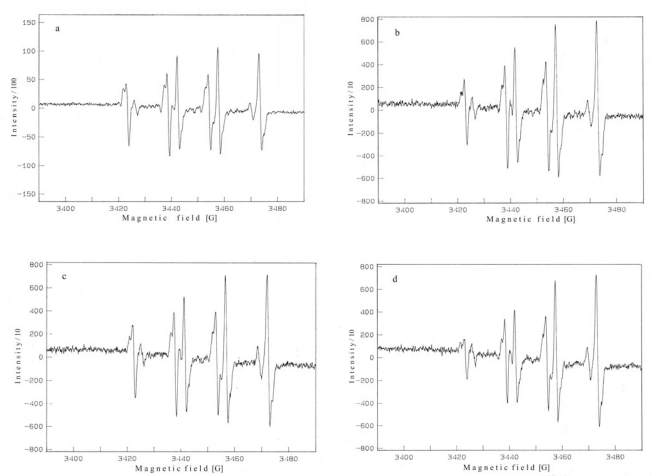
ESR spectra of DMPO-OH spin adducts: with no addition of extracts (blank) (a); the same as blank but with 0.3 mg/mL DMF solution of aqueous (b), ethanolic (c) and methanolic extract (d).

**Figure 2.**
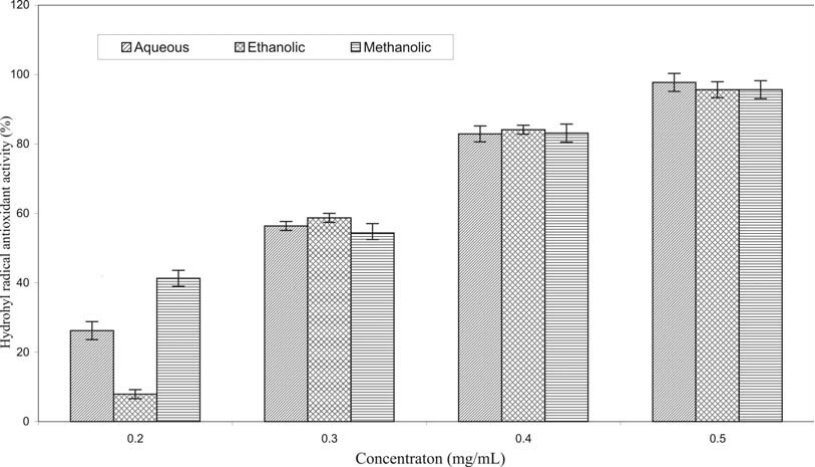
Antioxidant activity of different concentrations of aqueous, ethanolic and methanolic extracts of *Hieracium pilosella* L. on hydroxyl radical.

As shown in Figure 1a, the reaction of Fe^2+^ with H_2_O_2_ in the presence of spin trapping agent DMPO generated a 1:2:2:1 quartet of lines with hyperfine coupling parameters (*a*N = *a*H = 14.9G). The intensity of ESR signal, corresponding to the concentration of formed free radicals, was decreased in the presence of 0.30 mg/mL of aqueous (Figure 1b), ethanolic (Figure 1c) and methanolic extract (Figure 1d). The antioxidant activity of different concentrations of investigated extracts on hydroxyl radical is shown in Figure 2.

The investigation showed that the antioxidative activity increased with the increase of the concentration of all extracts. The concentration 0.5 mg/mL of aqueous, ethanolic and methanolic extract eliminated 97.78, 95.63 and 95.65% of the intensity of reference signal of DMPO-OH spin adduct, respectively.

The EC_50_ value is a widely used parameter to measure the free radical scavenging activity. A lower EC_50_ indicates a higher antioxidant activity [29]. EC_50_^OH^ values for aqueous, ethanolic and methanolic extract were: 0.279 ± 0.012, 0.283 ± 0.007 and 0.267 ± 0.005 mg/mL, respectively. Each value is mean ± SD of three measurements. EC_50_^OH^ values of the investigated extracts slightly differs depending on the solvent applied (EC_50_^OH^ value of methanolic extract is higher than EC_50_^OH^ of aqueous and ethanolic extract for 4.30 and 5.65%, respectively).

**Figure 3.**
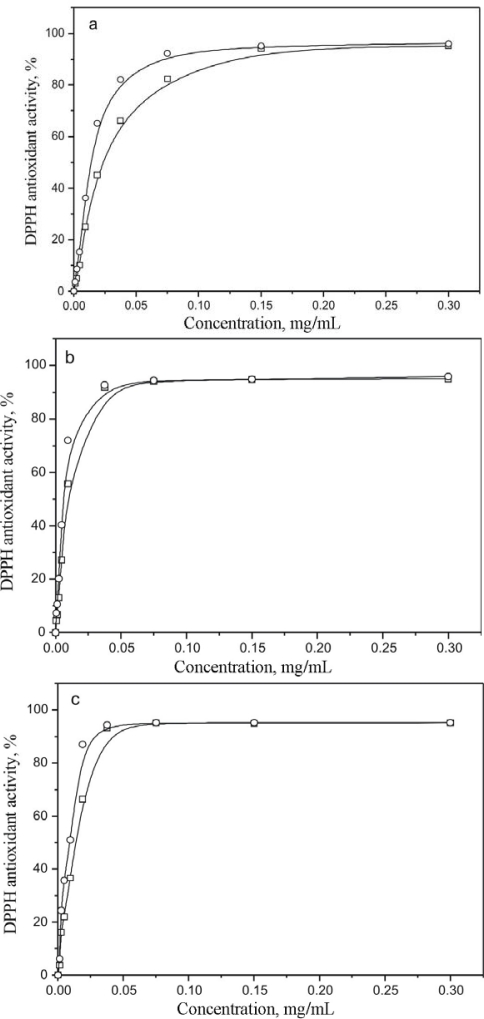
The antioxidant activity of different concentrations of aqueous (a), ethanolic (b) and methanolic (c) extract of *Hieracium pilosella* L. on DPPH radicals; (–□–) without incubation; (–○–) 20 min of incubation.

The DPPH^•^ test is based on the exchange of hydrogen atoms between the antioxidant and the stable DPPH^•^ free radical. Practically, the reaction brings about the reduction of DPPH^•^ radicals to the corresponding hydrazine, which is manifested by a color change from violet to yellow, which is monitored spectrophotometrically. The results for aqueous, ethanolic and methanolic extract are shown in Figures 3(a–c), respectively.

All the extracts show a higher DPPH^•^ radicals scavenging activity after incubation (20 min) with a free radical solution. DPPH^•^ antioxidant activity of aqueous, ethanolic and methanolic extracts increased with the increase of the concentration of all extracts (at concentrations ranging from 0.0012 to 0.30 mg/mL).

The DPPH^•^ antioxidant activity values of the aqueous, ethanolic and methanolic extracts at the concentration of 0.30 mg/mL were 96.10, 95.90 and 95.25% (20 min incubation time) and 95.20, 95.17 and 95.14% (without incubation), respectively. DPPH^•^ antioxidant activity of the investigated extracts slightly differs depending on the solvent applied (antioxidant activity of aqueous extract is higher than antioxidant activity of ethanolic and methanolic extract for 0.21 and 0.89%, respectively (20 min incubation time) and 0.03 and 0.06%, respectively (without incubation).

Degree values of DPPH^•^ radicals neutralization for all the three extracts do not differ significantly. The obtained results show that the time of incubation influences the % of free DPPH^•^ radical neutralization, but only up to a certain extract concentration. The incubation time has no influence on free DPPH^•^ radical neutralization for the extract concentrations higher than 0.075 mg/mL in the case of methanolic and ethanolic extracts, whereas this value in the aqueous extract is 0.15 mg/mL.

Unlike the examined extracts, the DPPH^•^ test performed without incubation showed that the standard BHT antioxidant did not reach the EC_50_ value at a concentration of 0.30 mg/mL. In the case of the test performed with a 20 minutes incubation, the BHT concentration necessary for reaching EC_50_ was 0.021 mg/mL. The obtained data show that the investigated extracts were better antioxidants than the BHT standard of the same concentration. A concentration of 0.30 mg/mL of aqueous, ethanolic and methanolic extract was less effective in scavenging hydroxyl radicals (56.35, 58.73 and 54.35%, respectively) in comparison with the DPPH radical scavenging activity (about 95% for all the extracts). In general, extracts with a high antioxidant activity showed a high phenolic content. Plant extracts with a high phenolic content also contained a high flavonoid content [29]. The EC_50_^DPPH^ values, the amount of total phenolic and flavonoids for all extracts are given in Table 3.

Based on the total phenolic content in the plant extracts, the selected parts can be divided into three ranges of GAE values. The lower, middle and higher ranges of total phenolic compounds were below 10, 10–20 and higher than 40 mg GAE/g dry weight of plant extract, respectively [29]. The results in the Table 3 show that all the investigated extracts have high phenolic and flavonoid contents. The total phenolic and flavonoid contents values do not significantly differ for aqueous, ethanolic and methanolic extracts.

## 3. Experimental Section

### 3.1. Plant Material

Whole plant (leaves and roots) of *Hieracium pilosella* L. (*Asteraceae*) was collected in Barje, Southeast Serbia, in June 2007 and identified by Professor Vlada Randjelovic at the Faculty of Mathematics and Natural Sciences of Nis. A voucher specimen (16 186 BEOU) is deposited in the herbarium of Botany and Botanical Garden, Faculty of Biology, University of Belgrade. The plant material was dried in the shade in an airy place and then stored in paperbags and kept at room temperature. Moisture content, determined by drying at 105 °C to constant weight, was 14.87%.

### 3.2. Chemicals and Reagents

HPLC grade acetonitrile (Merck, Darmstadt, Germany) and filtered bidistilled water were used for HPLC analysis. The solvents (methanol and ethanol) used for the extraction were from “Zorka” Farma (Scaron;abac, Serbia). Chlorogenic acid was obtained from Sigma-Aldrich (Steinheim, Germany). Apigenin-7-*O*-glucoside and umbelliferone were purchased from Extrasynthese (Genay, France). DPPH^•^ (1,1-diphenyl-2-picrylhydrazyl), DMPO (5,5-dimethyl-1-pyrroline-*N*-oxide), Folin-Ciocalteu reagent, gallic acid and rutin were obtained from Sigma Chemicals Co., (St. Louis, MO, USA). All other chemicals were of analytical reagent grade.

### 3.3. Extraction Method-Soxhlet Extraction

Dried, ground plant material (10 g) was extracted in a Soxhlet apparatus using the following solvents: water (the ratio of plant material to solvent was 1:25 m/v), 50% aqueous ethanol (the ratio of plant material to solvent was 1:15 m/v) and 80% aqueous methanol (the ratio of plant material to solvent was 1:20 m/v) [30,31]. The extraction was carried out at boiling temperature for 6 hours. The extracts obtained were evaporated under pressure at 50 °C to constant weight. The extracts were stored in the refrigerator for subsequents analysis.

### 3.4. Determination of Plant Extract Yield

The yield of evaporated dried extracts based on dry weight basis was calculated from the following equation:

$$\text{Yield}\;(g/100\;\text{g of dry plant material})=\left({\text{W}}_{1}\times 100\right)/{\text{W}}_{2}$$

where W_1_ was the weight of the extract after the solvent evaporation and W_2_ was the weight of the dry plant material.

### 3.5. HPLC Analysis

For the quantification of phenolic substances, the extracts were analyzed by HPLC under the following conditions: Apparatus: Agilent 110 Series, Waldborn, Germany; Column: Zorbax-Eclipse XDB-CN; 4.6 × 250 mm, 5 μm. Eluent: acetonitrile:water = 30:70 v/v. Flow rate: 1 mL/min. Injection volume: 20 μL. Temperature: 25 °C. Detection: diode-array detector (DAD), 205 nm. The quantitative determination of chlorogenic acid, apigenin-7-*O*-glucoside and umbelliferone was performed using external standards by the calibration curves of these compounds. The retention times and calibration curves (determined by HPLC method) of the investigated compounds in the *Hieracium pilosella* L. extracts are given in Table 4.

### 3.6. DPPH^•^ Assay

The capacity of a compound to scavenge free DPPH^•^ radicals is determined by the use of the so-called DPPH^•^ test [32–35]. The extracts obtained using the different solvents (10 mL) were evaporated on a rotary evaporator at 40 °C until dry, then dissolved in methanol and various concentrations of the methanolic extract solutions were prepared. A 1.0 mL of methanolic solution of DPPH^•^ radicals (3 × 10^−4^ mol/L) was added to 2.5 mL sample and measured immediately (without incubation) and after a 20 minute incubation period at room temperature. The absorbance of the samples was measured on a VARIAN UV–Vis Cary-100 Conc. spectrophotometer. The capacity of the scavenging free radicals was calculated as follows:

$${\mathrm{DPPH}}^{•}\;\mathrm{radicls}\;\mathrm{scavenging}\;\mathrm{capacity}\;(\%)=\left[1-\frac{\left(\mathrm{As}-\mathrm{Ab}\right)}{\mathrm{Ac}}\right]\cdot 100$$

where *As* is the sample absorbance at 517 nm of the sample of a methanolic solution of the extract treated with the DPPH^•^ radical solution, *Ab* is the blank absorbance at 517 nm of the blank methanol solution of the extract not treated with the DPPH^•^ radical solution and *Ac* is the control absorbance at 517 nm of the control solution of a pure, methanolic solution of DPPH^•^ radical (1.0 mL of DPPH^•^ radical of 3 × 10^−4^ mol/L concentration +2.5 mL of methanol). A decrease by 50% of the initial DPPH^•^ concentration was defined as the EC_50_. The EC_50_ value (mg/mL) was determined for all the extracts. BHT was used as the reference compound (EC_50_ = 0.021 mg/mL).

### 3.7. Hydroxyl Radical Assay

Hydroxyl radicals were obtained by the Fenton reaction in the following system: 0.2 mL 10 mM H_2_O_2_, 0.2 mL 10 mM FeCl_2_·4H_2_O and 0.2 mL 0.3 M DMPO as spin trap and 0.2 mL DMF (“blank”). The influence of the extracts on the formation and transformation of hydroxyl radicals was investigated by adding DMF solution of the extracts to the Fenton reaction system in the concentration range of between 0.2 to 0.5 mg/mL. ESR spectra were recorded after 5 min on an ESR spectrometer Bruker 300E (Rheinstetten, Germany) under the following conditions: field modulation, 100 kHz; modulation amplitude, 0.512 G; receiver gain, 5 × 10^5^; time constant, 81.92 ms; conversion time, 163.84 ms; centre field, 3440.00 G; sweep width, 100.00 G; *x*-band frequency, 9.64 GHz; power, 20 mW; and the temperature of 23 °C. The magnetic field scanning was calibrated using Fremy’s salt (peroxylamine disulphonate) [7]. Splitting constants were calculated from the computer-generated second derivatives of the spectra, after optimizing signal-to-noise ratios, and were verified by computer simulations. The scavenging effect of the extract was defined as:

$$\text{Scavenging effect}\;\left(\%\right)=\frac{h_{o}-h_{x}}{h_{o}}\cdot 100$$

where *h*_o_ and *h*_x_ are the height of the second peak in the ESR spectrum of DMPO-OH spin adduct of the blank and the sample, respectively. BHA was used as the reference compound (EC_50_ = 0.115 mg/mL).

### 3.8. Determination of Total Phenolic Content

The total phenolic content in the *H. pilosella* extracts was determined spectrophotometrically according to the Folin-Ciocalteu method [36] using galic acid as a standard (the concentration range: 0.025 to 0.5 mg/mL). The reaction mixture was prepared by mixing 1 mL of the methanolic solution (concentration 0.3 mg/mL), of the methanolic solution of the extract, 9 mL of distilled water, 1 mL of Folin-Ciocalteu’s reagent and 10 mL of 7% sodium carbonate. After the 90 minutes incubation at room temperature, the absorbance was determined spectrophotometrically at 765 nm. The total phenolic content was expressed as GAE in milligram per gram dry extract. The absorbance at 765 nm = 0.431 c_gallic acid_ (mg/mL) – 9.33 × 10^−3^, R^2^ = 0.9992.

### 3.9. Determination of Total Flavonoid Content

The total flavonoid content was determined according to the aluminium chloride colorimetric method [37]. Each plant extracts (2 mL, 0.3 mg/mL) in methanol were mixed with 0.1 mL of 10% aluminium chloride hexahydrate, 0.1 mL of 1 M potassium acetate and 2.8 mL of deionized water. After the 40 minutes incubation at the room temperature, the absorbance of the reaction mixture was determined spectrophotometrically at 415 nm. Rutin was chosen as a standard (the concentration range: 0.005 to 0.1 mg/mL) and the total flavonoid content was expressed as milligram RE per g of dry extracts. The absorbance at 415 nm = 14.171 c_rutin_ (mg/mL) + 0.0461, R^2^ = 0.9991.

### 3.10. Statistical Analysis

Results are expressed as the mean ± *S.D.* of three independent experiments. Student’s *t*-test was used for statistical analyses; P values > 0.05 were considered to be signficant.

## 4. Conclusions

In conclusion, this study indicates that the extracts obtained from the whole plant of *H. pilosella* L. have significant free radical scavenging activity on stable DPPH^•^ and high reactive hydroxyl radical. The data suggest that aqueous, ethanolic and methanolic extracts of *Hieracium pilosella* L. from Southeast Serbia are a potential source of natural antioxidants. Chlorogenic acid was detected in the highest quantities in all investigated extracts. More work should be done to characterize individual phenolic compounds of the extracts of *H. pilosella* L. in order to assign certain antioxidant effects to individual compounds of the resulting extracts.

## Figures and Tables

**Table 1.** The yields of the extracts obtained by the different solvents.

**Solvent**	**Total extract (g/100 g of dry plant material)**
Water	38.18 ± 1.13
Ethanol (50% v/v)	44.0 ± 1.03
Methanol (80% v/v)	42.33 ± 0 86

**Table 2.** The content of bioactive compounds in g per 100 g of the total dry extracts or the dry plant material.

	**Chlorogenic acid**		**Umbelliferone**		**Apigenin-7-*O*-glucoside**	

**Extract**	**Dry extract**	**Dry plant material**	**Dry extract**	**Dry plant material**	**Dry extract**	**Dry plant material**

Aqueous	52.30 ± 1.25	19.97 ± 0.85	1.69 ± 0.10	0.65 ± 0.06	0.21 ± 0.02	0.079 ± 0.08
Ethanolic	49.10 ± 1.20	21.60 ± 1.52	0.72 ± 0.15	0.31 ± 0.05	0.58 ± 0.03	0.250 ± 0.04
Methanolic	45.63 ± 1.10	19.20 ± 1.05	1.36 ± 0.2	0.58 ± 0.06	0.16 ± 0.04	0.068 ± 0.02

**Table 3.** Antioxidant activity, total phenolic content and total flavonoids of *H. pilosella* L. extracts.

**Extract**	**EC_50_^DPPH^, mg/mL**		**Total phenolic content, mgGAE/g**	**Total flavonoids, mgRE/g**
	**Without incubation**	**20 minutes incubation**		
Aqueous	0.023 ± 4×10^−4^	0.011 ± 2×10^−4^	239.59 ± 2.03	79.13 ± 0.47
Ethanolic	0.011 ± 5×10^−4^	0.007 ± 10^−4^	244.16 ± 2.15	82.18 ± 0.53
Methanolic	0.014 ± 3×10^−4^	0.009 ± 10^−4^	243.98 ± 2.14	81.52 ± 0.24

**Table 4.** Calibration curves and retention times of the investigated compounds in the *Hieracium pilosella* L. extracts (determined by HPLC method).

**Compound**	**Chlorogenic acid**	**Apigenin-7-*O*-glucoside**	**Umbelliferone**
Retention time, min	2.07	4.41	4.99
Concentration range, μg/mL	1 – 500	0.15 – 15	4 – 670
Calibration curve P[mAU]=q+r×c[mg/mL]^*^	q = 75.84 r = 30891.11	q = 60.08 r = 79938.97	q = 235.61 r = 153295.95
Correlation coefficient	0.9998	0.9997	0.9998

* P[mAU]: peak area; c[mg/mL]:concentration of the standard sample; q and r: constants
